# Effects of edge disorder on the stability of quantum oscillations in two-dimensional coupled systems

**DOI:** 10.1038/s41598-024-66391-5

**Published:** 2024-07-05

**Authors:** Yan-Yan Lu, Zhao-Nan Mu, Yu Huang, Gui-Rong Guo, Han-Hui Li, Shao-Jie Xiong, Jian-Xin Zhong

**Affiliations:** 1 School of Computer and Information Engineering, Guizhou University of Commerce, Guizhou, 550014 China; 2 Guizhou Power Grid Company Limited Guiyang Power Supply Bureau, Guizhou, 550001 China; 3 Institute for Quantum Science and Technology, Shanghai University, Shanghai, 200444 China; 4 School of Physics and Optoelectronics, Xiangtan University, Hunan, 411105 China

**Keywords:** Quantum diffusion, Electron oscillation, Coupled systems, Edge-disorder, Nanoscience and technology, Physics

## Abstract

This paper utilizes the theory of quantum diffusion to analyze the electron probability and spreading width of a wavepacket on each layer in a two-dimensional (2D) coupled system with edge disorder, aiming to clarify the effects of edge disorder on the stability of the electron periodic oscillations in 2D coupled systems. Using coupled 2D square lattices with edge disorder as an example, we show that, the electron probability and wavepacket spreading width exhibit periodic oscillations and damped oscillations, respectively, before and after the wavepacket reaches the boundary. Furthermore, these electron oscillations exhibit strong resistance against disorder perturbation with a longer decay time in the regime of large disorder, due to the combined influences of ordered and disordered site energies in the central and edge regions. Finally, we numerically verified the universality of the results through bilayer graphene, demonstrating that this anomalous quantum oscillatory behavior is independent of lattice geometry. Our findings are helpful in designing relevant quantum devices and understanding the influence of edge disorder on the stability of electron periodic oscillations in 2D coupled systems.

## Introduction

Layered materials, due to their weak interlayer van der Waals interactions, provide favorable conditions for the preparation of two-dimensional (2D) materials^1^. In 2004, Novoselov et al.^2^ isolated a monolayer of graphene through the mechanical exfoliation method, challenging the conventional belief that 2D crystalline materials cannot exist independently. Since then, 2D materials^3–5^ have become a focal point in the fields of condensed matter physics, materials science, and nanotechnology. With the research advances in 2D materials, few-layer graphene can be routinely obtained experimentally^6–12^. From 2018 to 2021, the emergence of superconductivity in bilayer^9,13^ and trilayer graphene^12^ has propelled the research on ultrathin quantum films to new heights. Ultrathin quantum films have garnered attention for their superior electrical properties^9–13^, but most research focuses on their crystal structure. However, an ultrathin quantum film initially made is likely to have irregular edges that could affect its performance. Even if initially perfect, there are still various types of disorder at the edges of such a finite size due to adsorption, lattice distortion, and interactions with the substrate. In practical preparation or application, inevitable irregularities such as roughness, undulations, and vacancies exist at the edges of quantum films. Edge disorder is an important factor affecting the electronic transport properties of materials^14^. Therefore, the study of the edge disorder effects on the stability of electrical properties in 2D coupled systems becomes crucial.

Disorders are almost unavoidable in real quantum materials, and they dramatically affect physical properties, especially the electron transport properties, regardless of whether the disorder is correlated^15–19^ or uncorrelated^20^. Anderson disorder^20,21^, as a prototypical model of uncorrelated disorder, has remained a key model in the field of disorder system research since its proposal. The electron localization^20,22–25^ and metal–insulator transition^26–28^ phenomena found in the Anderson model have drawn widespread attention in the field of condensed matter physics. Localization scaling theory^23^ predicts that in one- and two-dimensional (1D, 2D) systems with Anderson disorder in all site energies, the single-particle wave functions are always localized regardless of the disorder strength. Further studies have shown that this localization phenomenon also exists in quasi-one-dimensional^29^ and quasi-two-dimensional^27,30^ (quasi-1D, quasi-2D) systems.

Quantum dynamics^30,31^ are crucial for understanding the electronic transport properties in ordered and disordered systems. In quasi-1D and quasi-2D systems with Anderson disorder, although the electronic wave functions cannot extend to the boundaries, they can still hop regularly between chains or layers. In our previous research^29,30^, we used the method of quantum dynamics to predict that in low-dimensional coupled systems with mirror symmetry, periodic electronic oscillations exist. When all site energies are randomly disordered, these periodic electronic oscillations turn into damped oscillations with a continuously decreasing decay time as the degree of disorder increases. However, none of these studies have addressed the effects of edge-site energy disorder on the stability of quantum oscillations. Ultrathin quantum films often exhibit roughness, vacancies, and defects at edges due to cutting, material growth, adsorption, and other factors during materials fabrication. The random disorder of energies at the edge sites could affect the electronic transport properties. Therefore, it is interesting to study the stability of electron oscillations in 2D coupled systems with edge-site energy disorder.

This research is based on the Anderson model of 2D coupled systems. It employs the tight-binding approximation theory and the method of direct diagonalization of Hamiltonian matrix to solve both the static Schrödinger equation and the time-dependent Schrödinger equation. Additionally, the quantum diffusion theory is utilized to derive the time evolution of electron probability and wavepacket propagation width on each layer. We use a coupled bilayer of square lattices to explore the effects of edge-site energy disorder on the stability of the periodic electron oscillations in 2D coupled systems, and use the commonly studied material, bilayer graphene, to confirm the universality of these conclusions.

## Theory

**Figure 1:**
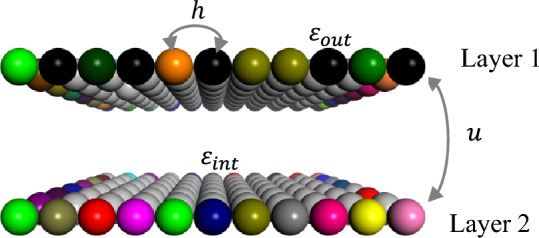
Schematic illustration of the edge-disordered 2D coupled system. $h$ and $u$ correspond to electron hopping parameters of intralayer and interlayer between nearest-neighbor sites, respectively. $\varepsilon_{int}$ and $\varepsilon_{out}$ represent the onsite energies in the center region(gray) and edge region(colored), respectively. The different colors indicate different types of atoms in the figure.

The proposed model is a finite-sized 2D coupled square lattices, where the edge site energies follow Anderson disorder, as illustrated in Fig. 1. During the numerical simulations, we use the single-electron tight-binding approximation theory, employing the method of direct matrix diagonalization and fixed boundary conditions to analyze the electron diffusion behavior in finite-sized 2D coupled systems. The Hamiltonian in the tight-binding form for a system with 2*N* sites is given by

1 $$H = H_{1} + H_{2} + H_{{{\text{int}}}}$$

where $H_{1}$, $H_{2}$ represent the sub-Hamiltonians of the layer 1, layer 2, and $H_{int}$ represents the interlayer Hamiltonian between the two-layers. Based on the tight-binding approximation theory, $H_{1}$, $H_{2}$ and $H_{int}$ can be described as follows

2 $$H_{1} = \mathop \sum \nolimits_{i = 1}^{N} \varepsilon_{i}^{\left( 1 \right)} \left| {1,i > < 1,i} \right| + \mathop \sum \nolimits_{ < i,j > }^{N} V_{ij}^{\left( 1 \right)} \left| {1,i > < 1,j} \right|$$

3 $$H_{2} = \mathop \sum \nolimits_{i = 1}^{N} \varepsilon_{i}^{\left( 2 \right)} \left| {2,i > < 2,i} \right| + \mathop \sum \nolimits_{ < i,j > }^{N} V_{ij}^{\left( 2 \right)} \left| {2,i > < 2,j} \right|$$

4 $$H_{{{\text{int}}}} = \mathop \sum \nolimits_{i = 1}^{N} V_{ii}^{{\left( {1,2} \right)}} \left| {1,i > < 2,i} \right| + \mathop \sum \nolimits_{i = 1}^{N} V_{ii}^{{\left( {2,1} \right)}} \left| {2,i > < 1,i} \right|$$

where $\left| {1,\left. i \right\rangle } \right.$, $\left| {2,\left. i \right\rangle } \right.$ represent the basis sets, and $\varepsilon_{i}^{\left( 1 \right)}$, $\varepsilon_{i}^{\left( 2 \right)}$ represent the onsite energies at the $i$-th site in layer 1 and layer 2, respectively. This paper only considers the effects of disorder in edge-site energies, so the energies of edge-sites (colored in Fig. 1, denoted as $\varepsilon_{out}$) are taken from random numbers in the interval [− $W$,$W$], referred to as the disordered region, while the energies of sites in the center region (gray in Fig. 1, denoted as $\varepsilon_{int}$) are set to 0, referred to as the ordered region, where $W$ represents the degree of disorder and corresponds to the periodic system with $W$ = 0. To simplify the calculation, only the hopping parameters of nearest-neighbor sites for intralayer hopping $V_{ij}^{\left( 1 \right)}$, $V_{ij}^{\left( 2 \right)}$ and interlayer hopping $V_{ii}^{{\left( {1,2} \right)}}$, $V_{ii}^{{\left( {2,1} \right)}}$ are considered, with $V_{ij}^{\left( 1 \right)} = { }V_{ij}^{\left( 2 \right)}$ = $h$ and $V_{ii}^{{\left( {1,2} \right)}} = { }V_{ii}^{{\left( {2,1} \right)}} = u$, where $i$ and ${ }j$ represent the $i$- and $j$-th sites. During the numerical simulations, all physical quantities are expressed in dimensionless units. The lattice constant and the intralayer hopping strength are normalized with $a$ = 1, $h$ = 1 and $u = 1$, respectively. The interlayer hopping strength $u$ and the degree of disorder $W$ are treated as modulated variables for tuning quantum diffusion.

The electron eigen-states of the 2D coupled system can be obtained by the following static Schrödinger equation

5 $$H\left| {\left. \psi \right\rangle } \right. = E\left| {\left. \psi \right\rangle } \right.$$

where $E$ and $\left| {\left. \psi \right\rangle } \right.$ are the eigen-energy and eigen-wavefunction, respectively. If layer 1 and layer 2 have a mirror-symmetric structure, regardless of edge-disordered or not, $H_{1}$ and $H_{2}$ are iso-spectral with the same spectrum of electron eigen-energy $e_{l}$ $\left( {l = 1,2, \ldots N} \right)$, and their corresponding electron wavefunctions $\left| \varphi \right.\left. {_{l}^{\left( 1 \right)} } \right\rangle$, $\left| \varphi \right.\left. {_{l}^{\left( 2 \right)} } \right\rangle$ are respectively given by

6 $$\left| {\varphi _l^{(1)}} \right\rangle = \sum\limits_{j = 1}^N {{C_{lj}}} \left| {1,j} \right\rangle,\quad\left| {\varphi _l^{(2)}} \right\rangle = \sum\limits_{j = 1}^N {{C_{lj}}} \left| {2,j} \right\rangle,$$

Projecting $\left| {\left. \psi \right\rangle } \right.$ to the basis set formed by $\left| \varphi \right.\left. {_{l}^{\left( 1 \right)} } \right\rangle$ and $\left| \varphi \right.\left. {_{l}^{\left( 2 \right)} } \right\rangle$, one has

7 $$\left| {\left. \psi \right\rangle } \right. = \mathop \sum \limits_{l = 1}^{N} \left| {\left. {\varphi_{l}^{\left( 1 \right)} } \right\rangle } \right.\left\langle \varphi \right._{l}^{\left( 1 \right)} \left| {\left. \psi \right\rangle + \mathop \sum \limits_{l = 1}^{N} \left. {\left| {\varphi_{l}^{\left( 2 \right)} } \right.} \right\rangle \left\langle {\varphi_{l}^{\left( 2 \right)} } \right.\left| {\left. \psi \right\rangle } \right.} \right.$$

Substituting Eq. (7) into Eq. (5) and using Eqs. (1)–(4), one can derive and describe the electron wavefunctions and eigen-energies of the 2D coupled system as follows

8 $$E_{l}^{ \pm } = e_{l} \pm u$$

9 $$\left| {\left. {\psi_{l}^{ \pm } } \right\rangle } \right. = \frac{1}{\sqrt 2 }\left( {\left| {\left. {\varphi_{l}^{\left( 1 \right)} } \right\rangle \pm \left| {\left. {\varphi_{l}^{\left( 2 \right)} } \right\rangle } \right.} \right.} \right)$$

The dynamics of an electron wavepacket in the 2D coupled system are governed by the time-dependent Schrödinger equation

10 $$i\frac{d}{dt}\left. {\left| {{\Psi }\left( t \right)} \right.} \right\rangle = H\left. {\left| {{\Psi }\left( t \right)} \right.} \right\rangle$$

where $\left| {{\Psi }\left. {\left( t \right)} \right\rangle } \right. = \left( {{\Psi }_{1} ,{{ \Psi }}_{2} ,{ } \ldots {\Psi }_{2N} } \right)^{T}$ represents the electron wavefunction at time $t$, with ${\Psi }_{n} \left( t \right)$ being the coefficient of $\left. {\left| {{\Psi }\left( t \right)} \right.} \right\rangle$ corresponding to site $n$. If the initial state of electron wavepacket is at position $m$ in layer 1, then $\left| {{\Psi }\left. {\left( t \right)} \right\rangle } \right._{t = 0} = \left| {1, \left. m \right\rangle } \right.$, and thus

11 $$\left| {{\Psi }\left. {\left( t \right)} \right\rangle } \right. = \frac{1}{\sqrt 2 }\mathop \sum \limits_{l = 1}^{N} C_{lm}^{*} e^{{ - ie_{l} t}} \left( {e^{ - iut} \left| {\psi \left. {_{l}^{ + } } \right\rangle + e^{iut} \left| {\left. {\psi_{l}^{ - } } \right\rangle } \right.} \right.} \right)$$

where, $C_{lm}^{*}$ is the complex conjugate of $C_{lm}$. Projection of $\left. {\left| {{\Psi }\left( t \right)} \right.} \right\rangle$ to the basis set of layer 1 and layer 2 leads to

12a $${\Psi }_{n}^{\left( 1 \right)} \left( t \right) = \left\langle {1,n} \right.\left. {\left| {{\Psi }\left( t \right)} \right.} \right\rangle = \cos (ut){\Psi }_{nm}^{\left( s \right)} \left( t \right)$$

12b $${\Psi }_{n}^{\left( 2 \right)} \left( t \right) = \left\langle {2,n} \right.\left| {\left. {{\Psi }\left( t \right)} \right\rangle } \right. = - i\sin (ut){\Psi }_{nm}^{\left( s \right)} \left( t \right)$$

where ${\Psi }_{nm}^{\left( s \right)} \left( t \right) = \mathop \sum \nolimits_{l = 1}^{N} C_{{l{ }n}} C_{lm}^{*} e^{{ - ie_{l} t}}$ is the wavefunction of the single isolated layer with initial distribution of wavepacket^31^. The probability of finding electron at the *n-*th site on layer 1 and layer 2 follows

13a $$P_{n}^{\left( 1 \right)} \left( t \right) = {\text{cos}}^{2} \left( {ut} \right)P_{nm}^{\left( s \right)} \left( t \right)$$

13b $$P_{n}^{\left( 2 \right)} \left( t \right) = {\text{sin}}^{2} \left( {ut} \right)P_{nm}^{\left( s \right)} \left( t \right)$$

where $P_{nm}^{\left( s \right)} \left( t \right) = \left( {\mathop \sum \nolimits_{l = 1}^{N} C_{{l{ }n}} C_{lm}^{*} e^{{ - ie_{l} t}} } \right)^{2} { }$ is the probability of finding electron at site *n* on the single isolated layer.

As the probability of finding electron on layer 1 is given by $P_{1} \left( t \right) = \mathop \sum \nolimits_{n = 1}^{N} P_{n}^{\left( 1 \right)} \left( t \right){ }$, on layer 2 is given by $P_{2} \left( t \right) = \mathop \sum \nolimits_{n = 1}^{N} P_{n}^{\left( 2 \right)} \left( t \right)$, and taking into account the rule of $P_{1} \left( t \right) + P_{2} \left( t \right) = 1$, one has

14a $$P_{1} \left( t \right) = {\text{cos}}^{2} \left( {ut} \right) = 0.5 + 0.5\cos (2ut)$$

14b $$P_{2} \left( t \right) = {\text{sin}}^{2} \left( {ut} \right) = 0.5 - 0.5\cos (2ut)$$

From Eqs. (13a) and (13b), one can also derive that the spreading widths of electron wavepacket $d_{1} \left( t \right)$ on layer 1, defined as $d_{1}^{2} \left( t \right) = \mathop \sum \nolimits_{n = 1}^{N} P_{n}^{\left( 1 \right)} \left( t \right)(n - m)^{2}$, and $d_{2} \left( t \right)$ on layer 2, defined as $d_{2}^{2} \left( t \right) = \mathop \sum \nolimits_{n = 1}^{N} P_{n}^{\left( 2 \right)} \left( t \right)\left[ {\left( {n - m} \right)^{2} + a^{2} } \right]$, are given by

15a $$d_{1}^{2} \left( t \right) = {\text{cos}}^{2} \left( {ut} \right)d_{nm}^{2} \left( t \right)$$

15b $$d_{2}^{2} \left( t \right) = s{\text{in}}^{2} \left( {ut} \right)\left[ {d_{nm}^{2} \left( t \right) + a^{2} } \right]$$

with

16 $$d_{nm}^{2} \left( t \right) = \mathop \sum \limits_{n = 1}^{N} P_{nm}^{\left( s \right)} \left( t \right)(n - m)^{2}$$

where $d_{nm}^{2} \left( t \right)$ is the spreading width of wavepacket on isolated single layer without coupling, and $n$, $m$ represent the planar positions of the $n$-th and $m$-th sites, respectively. The spreading width of electron wavepacket of the 2D coupled system is described by $d\left( t \right) = \sqrt {d_{1}^{2} \left( t \right) + d_{2}^{2} \left( t \right)}$. According to the theory of quantum diffusion, the relationship between $d\left( t \right)$ and time $t$ follows $d\left( t \right) \propto t^{\beta }$ with $0 \le \beta \le 1$, where $\beta$ = 0, 0.5, 1.0 corresponds to localization, normal diffusion, and ballistic diffusion, respectively, 0 < $\beta$ < 0.5 and 0.5 < $\beta$ < 1, correspond to sub-diffusion and super-diffusion. The analysis shows that, only in the special case of strong localization does the localization length become close to the lattice spacing, $d_{nm} \left( t \right){ }\sim { }a$. At this moment, $d_{1} \left( t \right)$ and $d_{2} \left( t \right)$ exhibit different amplitudes of oscillation. However, in general, $d_{nm} \left( t \right){ }$ is much larger than $a$, which means that, for large time $d_{nm} \left( t \right)$≈ *d*(*t*). In this case, the spreading width of wavepacket or the oscillation length on each layer can be given by

17a $$d_{1} \left( t \right) = d\left( t \right)|\cos (ut)|$$

17b $$d_{2} \left( t \right) = d\left( t \right)|\sin (ut)|$$

By defining the spreading width of wavepacket ratio $\overline{d}_{k} \left( t \right) = d_{k} \left( t \right)/d\left( t \right)$ one can obtain

18a $$\overline{d}_{1}^{2} \left( t \right) = {\text{cos}}^{2} \left( {ut} \right) = 0.5 + 0.5\cos (2ut)$$

18b $$\overline{d}_{2}^{2} \left( t \right) = sin^{2} \left( {ut} \right) = 0.5 - 0.5\cos (2ut)$$

Equations (14a), (14b), and Eqs. (18a), (18b) clearly indicate that both the probability $P_{k} \left( t \right)$ of finding electron on $k$-th layer, and the square of the spreading width of wavepacket ratio $\overline{d}_{k}^{2} \left( t \right)$, exhibit a standard periodic oscillation with frequency $f = u/\pi$, which is determined only by the interlayer hopping strength. This means that the 2D coupled system with a mirror-symmetric structure, regardless of order or edge-disorder, exhibits a periodic oscillation in both transverse and longitudinal directions with the same frequency, due to the iso-spectra of two-layers, which are independent of system size and type of intralayer potentials.

## Numerical results

The numerical results for the edge-disordered bilayer square lattices and the bilayer graphene are as follows. In the numerical calculations, the initial position of the electron wavepacket is located at the central site of layer 1. Considering both the accuracy of conclusions and the statistical significance, the results presented below are from the average of 20 different random samples, each with a system size of 2$N$ = 10,082 for the bilayer square lattices and 2$N$ = 7688 for the bilayer graphene. Since $P_{1} \left( t \right) + P_{2} \left( t \right) = 1$ and $\overline{d}_{1}^{2} \left( t \right) + \overline{d}_{2}^{2} \left( t \right) = 1$, we solely focus on the analysis of $P_{1} \left( t \right)$ and $\overline{d}_{1}^{2} \left( t \right)$.

**Figure 2:**
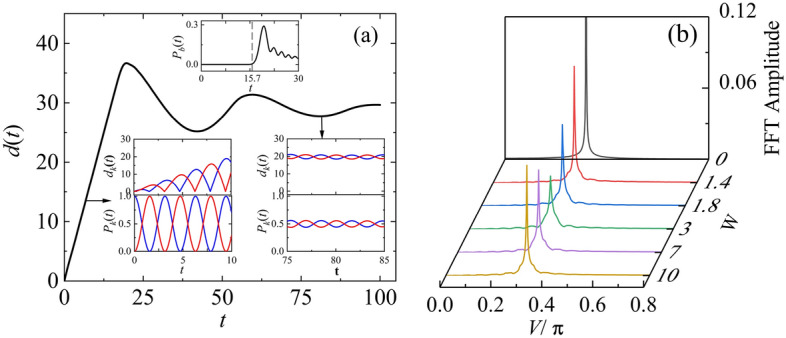
Electron diffusion in an edge-disordered bilayer square lattice with $2N = 71 \times 71 \times 2$, $h$ = 1, $u$ = 1, $\varepsilon_{int}$ = 0, $\varepsilon_{out}$ ∈ [$- W,W$]. (**a**) The spreading width of electron wavepacket $d\left( t \right)$ in the edge-disordered 2D coupled system with $W$ = 3. The insets show the probability of finding electron at the edge-sites $P_{b} \left( t \right)$, the spreading width $d_{k} \left( t \right)$ and the probability $P_{k} \left( t \right)$ of find electron in the $k$-th layer, where $k$ = 1,2 corresponds to the blue and red curve, respectively. (**b**) FFT frequency spectrum of probability $P_{1} \left( t \right)$ as a function of $W$.

Figure 2 illustrates the electron diffusion in an edge-disordered coupled system of bilayer square lattice (BSL). In Fig. 2a, it can be observed that the spreading width of wavepacket, denoted as $d\left( t \right)$, exhibits linear increase with time before the wavepacket reaches the boundary (*t* < 15.7), and the increase ceases after the wavepacket reaches the boundary (*t* > 15.7), which is similar to the case without edge disorder^30^. The dashed line in the $P_{b} \left( t \right)$ inset marks the time when the wavepacket reaches the boundary, and the time intervals 0 ≤ t ≤ 10 and 75 ≤ t ≤ 85 of the $d_{k} \left( t \right)$ and $P_{k} (t$) insets correspond to the scenarios before and after the wavepacket reaches the boundary. From the figures of $d_{k} \left( t \right)$ and $P_{k} (t$) sets one can see that the amplitude of the electron oscillations significantly decreases after the wavepacket reaches the boundary. In Fig. 2b, we present the Fast Fourier transformation (FFT) spectrum of the electron probability on layer 1, $P_{1} \left( t \right)$, under different disorder strength $W$. The figure shows that, as $W$ increases, $P_{1} \left( t \right)$ maintains a single-frequency signal pattern, and the signal strength undergoes a transition from a decrease to an increase at $W$ = 3, marking a difference from traditional Anderson disordered systems^30^.

Numerical analysis reveals that in the edge-disordered 2D coupled system, before the wavepacket reaches the boundary, both the electron probability $P_{k} \left( t \right)$ and the square of the spreading width of the wavepacket ratio $\overline{d}_{k}^{2} \left( t \right)$ exhibit periodic oscillations, similar to those observed in periodic systems, following the Eqs. (14) and (18). After the wavepacket reaches the boundary, both $P_{k} \left( t \right)$ and $\overline{d}_{k}^{2} \left( t \right){ }$ display damped periodic oscillations similar to traditional Anderson disordered systems, as described by the following

19 $$P_{k} \left( t \right) = \overline{d}_{k}^{2} \left( t \right) = 0.5 \pm 0.5e^{{ - \frac{{t - t_{b} }}{{t_{0} }}}} \cos (2V\left( {t - t_{b} } \right)){ }\left( {k = 1,2} \right)$$

where, $t_{0}$ represents the decay time, $V/\pi$ is the frequency parameter, and $t_{b}$ is the time when the peak of the wavepacket reaches the boundary.

**Figure 3:**
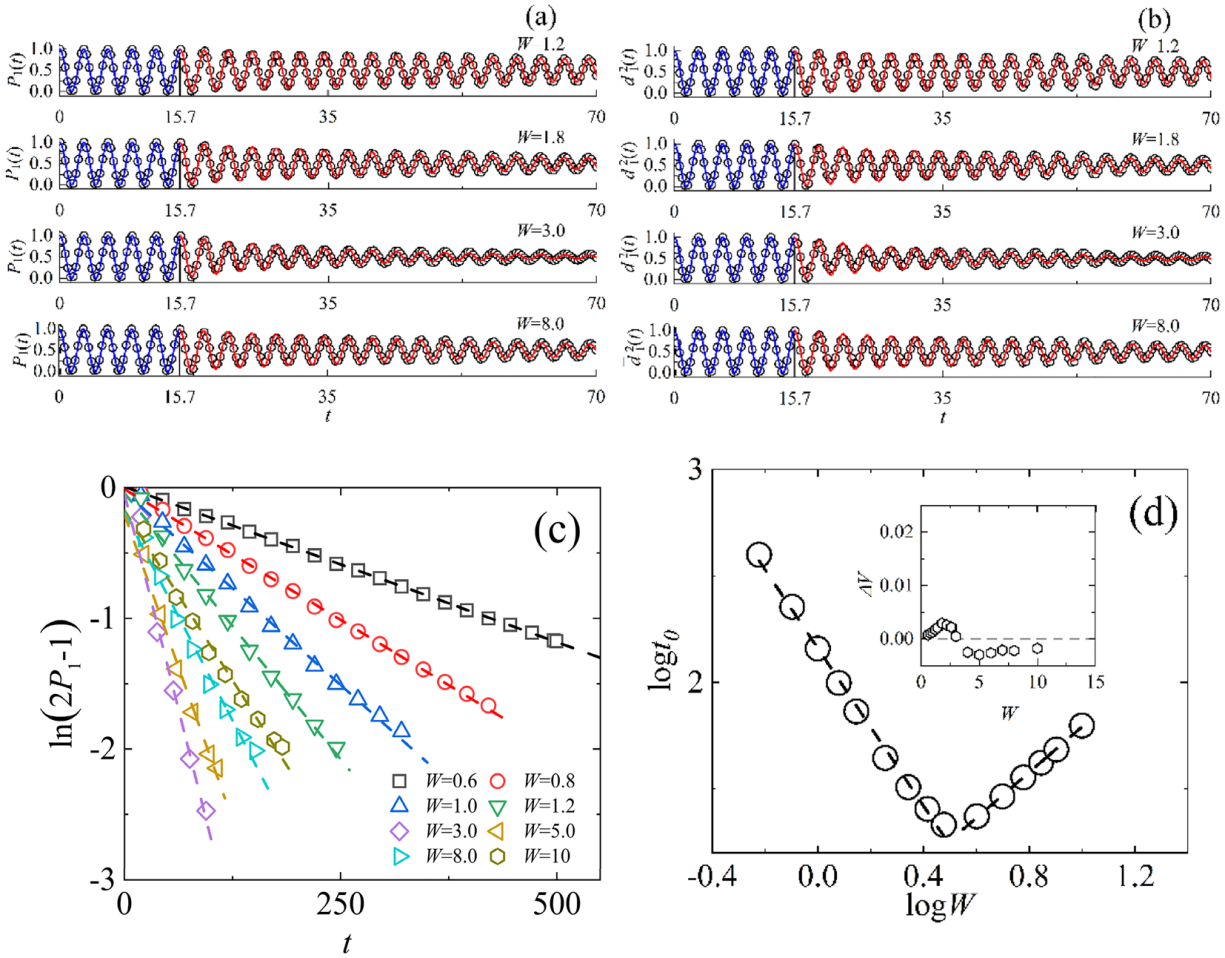
Electron oscillations of probability $P_{1} \left( t \right)$ and the square of wavepacket spreading width ratio $\overline{d}_{1}^{2} \left( t \right)$ in an edge-disordered bilayer square lattice, with $2N = 71 \times 71 \times 2$, $h$ = 1, $u$ = 1, $\varepsilon_{int}$ = 0, $\varepsilon_{out}$ ∈ [$- W,W$]. (**a**) and (**b**) Show the numerical results (open circles) of $P_{1} \left( t \right)$ and $\overline{d}_{1}^{2} \left( t \right)$ for $W = 1.2, {1}{\text{.8, 3}}{.0, 8}{\text{.0}}$, and the corresponding fitting results based on Eq. (19) with red lines($t$ > 15.7), and both Eq. (14a) and Eq. (18a) with blue line(t < 15.7). (**c**) The numerical results of $\ln \left( {2P_{1} - 1} \right){ }$(symbols) as a function of $t$, and the corresponding fitting results (dash lines) with linear equation $\ln (2P_{1} - 1) = t/t_{0}$, where $P_{1}$ corresponds to the amplitude of oscillations of $P_{1} \left( t \right)$. (**d**) Values of log $t_{0}$ versus log $W$ (symbols) and the corresponding linear fitting (dashed line) with log $t_{0}$ ~ log $W$, as well as values of $\Delta V$ versus $W$ (inset), where $\Delta V = V - V_{0}$, and $V_{0}$ = $u$ is the parameter of the bilayer square lattice without disorder.

Figure 3 illustrates the electron oscillations of probability $P_{1} \left( t \right)$ and the square of wavepacket spreading width ratio $\overline{d}_{1}^{2} \left( t \right)$ in an edge-disordered bilayer square lattice. Figure 3a and b indicate that, before the wavepacket reaches the boundary ($t$ ≤ 15.7), both $P_{1} \left( t \right)$ and $\overline{d}_{1}^{2} \left( t \right)$ exhibit periodic oscillations, following Eq. (14a) and Eq. (18a), respectively. After the wavepacket reaches the boundary ($t$ > 15.7), these peaks of periodic oscillations undergo exponential decay over time and become damped oscillations, which can be described as Eq. (19). More importantly, the decay rate $1/t_{0}$ of these damped oscillations exhibits a transition from acceleration to deceleration as $W$ increases, occurring at $W$ = 3. In the two figures, $W = {1}{\text{.2}},{ 1}{\text{.8}},{ 3}{\text{.0}},{ 8}{\text{.0}}$ correspond to $t_{0} = {99}{\text{.4}},{ 44}{\text{.2}},{ 21}{\text{.5}},{ 48}{\text{.4}}$ and $V = 1.0{0171},{ }1.0{0289},{ }1.0{0047},{ 0}{\text{.99779}}$, respectively. Here, $t_{b}$ = 15.7 which can be inferred from $P_{b} \left( t \right)$. The fitting parameters $t_{0}$, $t_{b}$, $V$ for $\overline{d}_{1}^{2} \left( t \right)$ are the same as those for $P_{1} \left( t \right)$. According to Eq. (19), one can infer that $\ln (2P_{1} - 1) = t/t_{0}$. Figure 3c presents the numerical results of $\ln (2P_{1} - 1)$ as a function of $t$ for different $W$, and their corresponding linear fits of the linear equation $\ln (2P_{1} - 1) = t/t_{0}$, where $W$ = 0.6, 0.8, 1.0, 1.2, 3.0, 5.0, 8.0, 10 correspond to $t_{0}$ = 397.84, 226.66, 144.19, 99.45, 21.46, 29.18, 48.43, 62.76. To obtain precise fitting results, we initially estimate the value of $t_{0}$ by performing a test fit using numerical data with large time values ($t > t_{0}$). Subsequently, we extract the refined fitting parameters $t_{0}$ by conducting repeated fits using data during $0 < t < t_{0}$, until the slope $1/t_{0}$ remains constant. In this figure we can see a significant reversal in the fitting slope at $W$ = 3. As $W$ increases, the slope increases for $W$ < 3 and decreases for $W$ > 3. By substituting $t_{0}$ into Eq. (19), the frequency parameter $V$ can be determined by fitting the data for $0 < t < t_{0}$ of $P_{1} \left( t \right)$. Figure 3d presents the numerical results of $t_{0}$ with different $W$ in log–log and their linear fitting with function ${\text{log}}t_{0} \sim {\text{log }}W$, which clearly indicate that $t_{0} \sim W^{\alpha }$, with α = *-*1.85 for $W$ < 3, and α = 1.07 for $W$ ≥ 3, where $t_{0}$ is the decay time of the electron oscillation. This anomalous quantum diffusion phenomenon suggests that, in edge-disordered 2D coupled systems, the damped oscillations will improve with the degree of disorder increases when $W$ exceeds a critical value, larger disorder leads to a longer decay time. The inset shows that the frequency $V/\pi$ only undergoes a slight change with different degrees of disorder, $\Delta V = V - V_{0} \approx 0$. All of those indicate that, compared to traditional disordered 2D coupled systems^30^, the electron oscillations in edge-disordered 2D coupled systems are more stable against disorder.

**Figure 4:**
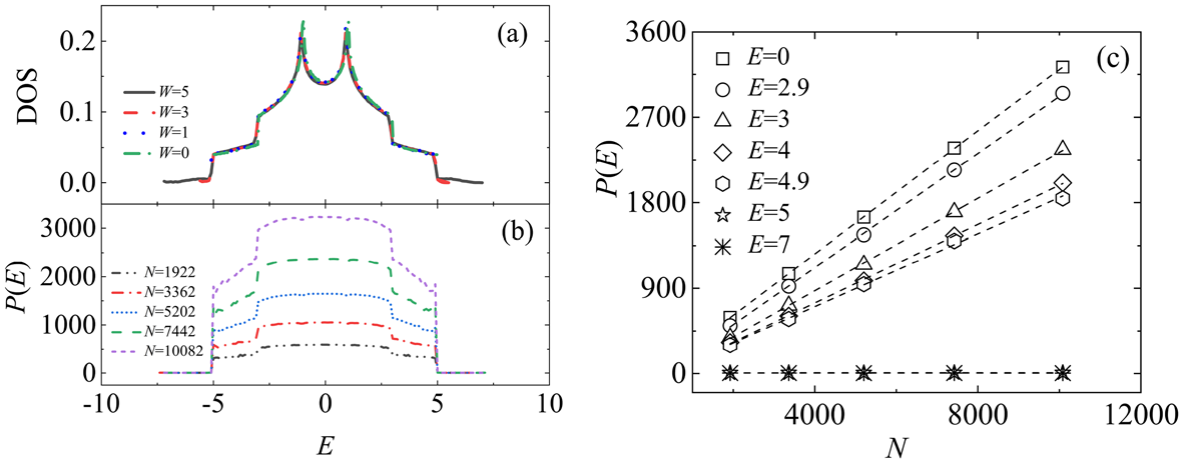
The density of states (DOS) and participation number $P\left( E \right)$ in the edge-disordered bilayer square lattice with $h$ = 1, $u$ = 1, $\varepsilon_{int}$ = 0, $\varepsilon_{out}$ ∈ [$- W,W$]. (**a**) DOS as a function of $W$ with system size $2N = 71 \times 71 \times 2$. (**b**) $P\left( E \right)$ as a function of $2N$ for systems with $W$ = 5. (**c**) The scaling behavior of $P\left( E \right)$ for systems of $2N$ = 1922, 3362, 5202, 7442, 10,082, with $W$ = 5, where symbols are the numerical results, and dashed lines represent the fitting lines of the function $P\left( E \right) \propto (2N)^{\gamma }$, with $\gamma = 1$ for *|E|*< 5, and $\gamma = 0$ for *|E|*$>$ 5.

The anomalous quantum diffusion behavior in edge-disordered coupled systems results from the combined influence of disordered edge-site energies and ordered site energies in the central regions. The degree of electron localization in quantum diffusion can be described by the particle participation number, which is defined as $P(E)^{{ - {1}}} = \mathop \sum \nolimits_{n} \phi \left( {E,\;\vec{r}_{n} } \right)^{4}$, where $\phi \left( {E, \;\vec{r}_{n} } \right)$ is the wave function of the state $E$. Here, $P\left( E \right)$ is related to the system size 2*N* as $P\left( E \right) \propto \left( {2N} \right)^{\gamma }$, with $\gamma = 1$ for extended states, $\gamma = 0$ for localized states, and 0 < γ < 1 for critical states. Figure 4 shows that, the energy spectrum of the edge-disordered coupled system is divided into a stable central region and two extended localized tails, separated by the critical energy levels $E_{c}$ =  ± 5, due to the influence of the ordered central part and the disordered edges. Figure 4a displays the density of states (DOS) for different values of $W$. Figure 4a shows that the DOS exhibits the same behavior as a bilayer periodic system for any values of $W$ in the band center region (*|E|*< 5), while its band tails ($\left| E \right|$> 5) continuously expand as $W$ increases, similar to those in a traditional disordered bilayer^27^. We take the edge-disordered system with $W$ = 5 as an example to demonstrate the relationship between $P\left( E \right)$ and the system size $2N$, as shown in Fig. 4b and c. From Fig. 4b, it is evident that the numerical values of $P\left( E \right)$ noticeably increase as $2N$ increases for $\left| E \right|$< 5, but independent of $2N$ for $\left| E \right|$> 5. The detailed analysis in Fig. 4c shows that, there is a significant mobility edge at $\left| E \right|$= ± 5, dividing the energy spectrum into a ballistic central region ($\left| E \right|$< 5, $\gamma$ = 1) and two localized tails ($\left| E \right|$> 5, $\gamma$ = 0). Considering the symmetry of the $P\left( E \right)$ spectrum and for the sake of graphical clarity, the figure only shows several typical energy levels for $E$ > 0.

Consider a bilayer square lattice system with $2N$ sites. By solving the Schrödinger equation, the electron wavefunction component at the *n-*th site at time* t* can be given by

$$\psi_{n} \left( t \right) = \mathop {\Sigma }\limits_{j} \phi_{0}^{*} \left( {E_{j} } \right)\phi_{n} \left( {E_{j} } \right)\exp ( - iE_{j} t).$$

Here, $E_{j} \left( {j = 1,2,3,4, \ldots 2N} \right)$ are the eigen-energies, $\phi_{n} \left( {E_{j} } \right)$ is the eigen-wavefunctions corresponding to $E_{j}$ at *n-*th site, where $\phi_{0}^{*} \left( {E_{j} } \right)$ is the complex conjugate of $\phi_{n} \left( {E_{j} } \right)$. Therefore, the probability of finding electron at *n-*th site at time *t* can be expressed as $P_{n} \left( t \right) = \left| {\psi_{n} \left( t \right)} \right|^{2}$. According to the previous discussion, the electron wavefunction in an edge-disordered bilayer system encompasses both extended states and localized states. Therefore, the probability of finding electron at *k-*th (*k* = 1, 2) layer can be expressed as

$$P_{k} \left( t \right) = \mathop \sum \limits_{n = 1}^{N} P_{n}^{\left( k \right)} \left( t \right) = \mathop \sum \nolimits_{NO} P_{no}^{\left( k \right)} \left( t \right) + \mathop \sum \nolimits_{ND} P_{nd}^{\left( k \right)} \left( t \right).$$

Here, $\mathop \sum \nolimits_{NO} P_{no}^{\left( k \right)} \left( t \right)$ represents the probability of finding electron in the central region with ordered sites ($\varepsilon_{int}$ = 0) in the *k-*th layer, and $\mathop \sum \nolimits_{ND} P_{nd}^{\left( k \right)} \left( t \right)$ represents the probability of finding electron at the edge disordered sites ($\varepsilon_{out} \in \left[ { - W, W} \right])$ in the *k-*th layer. According to the theory of electron localization, as *W* increases, the edge wavefunctions become more and more localized. In the case of large disorder, $\mathop \sum \nolimits_{ND} P_{nd}^{\left( k \right)} \left( t \right)$ approaches to a constant, and thus $P_{k} \left( t \right) \to \mathop \sum \nolimits_{NO} P_{no}^{\left( k \right)} \left( t \right)$ + constant, which means that the electron oscillation behavior will gradually approaches that in a bilayer periodic system. This is also the reason why, in the edge-disordered coupled system, when $W$ exceeds a critical value, larger disorder leads to longer decay times in damped oscillations. This anomalous behavior is similar to the crossover of quantum diffusion in the bilayer system with order–disorder separation^26–28,32^.

**Figure 5:**
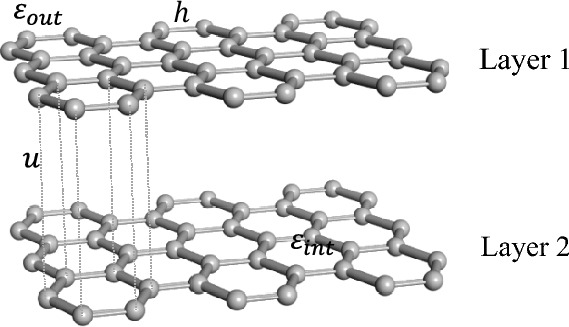
Schematic illustration of bilayer graphene of AA-stacking(the upper atom is directly above the lower atom). Parameters $h$ and $u$ correspond to nearest-neighbor hopping energy of intralayer and interlayer, respectively. $\varepsilon_{int}$ and $\varepsilon_{out}$ are the site energies in the central ordered region and in the edge disordered region, respectively.

However, compared to the square lattices, hexagonal lattices are more prevalent in 2D materials. In the following sections, we will take a bilayer graphene of AA-stacking, shown in Fig. 5, as an example to validate the universality of electron oscillations in edge-disordered 2D coupled systems. The system size of the bilayer graphene used for numerical computation is $2N = {7688}$, with $x = 31 \times 2a$, $y = 31 \times \frac{\sqrt 3 }{2}a$, $z = d$, where $a$ is the lattice spacing equal to 0.142 nm^33^, and $d$ is the distance between the layers equal to 0.335 nm^33^. Additionally, the nearest-neighbor hopping energies $h$ is $- 2.7\;\;{\text{eV}}$^33,34^. To simplify the numerical calculation, all physical parameters mentioned above are used in dimensionless form. Here, $a$, $d$, and $h$ are set to 0.142, 0.335, -2.7, respectively. The nearest-neighbor interlayer hopping parameter $u$ is set to 1, the site energies of the central ordered region are set to $\varepsilon_{int}$ = 0, while energies of the disordered sites at the edge are set to $\varepsilon_{out} \in$[$- W, W$]. The numerical results presented below are from average of 20 different disordered configurations to ensure reliable results. The initial location of the electron is at the center site of layer 1.

**Figure 6:**
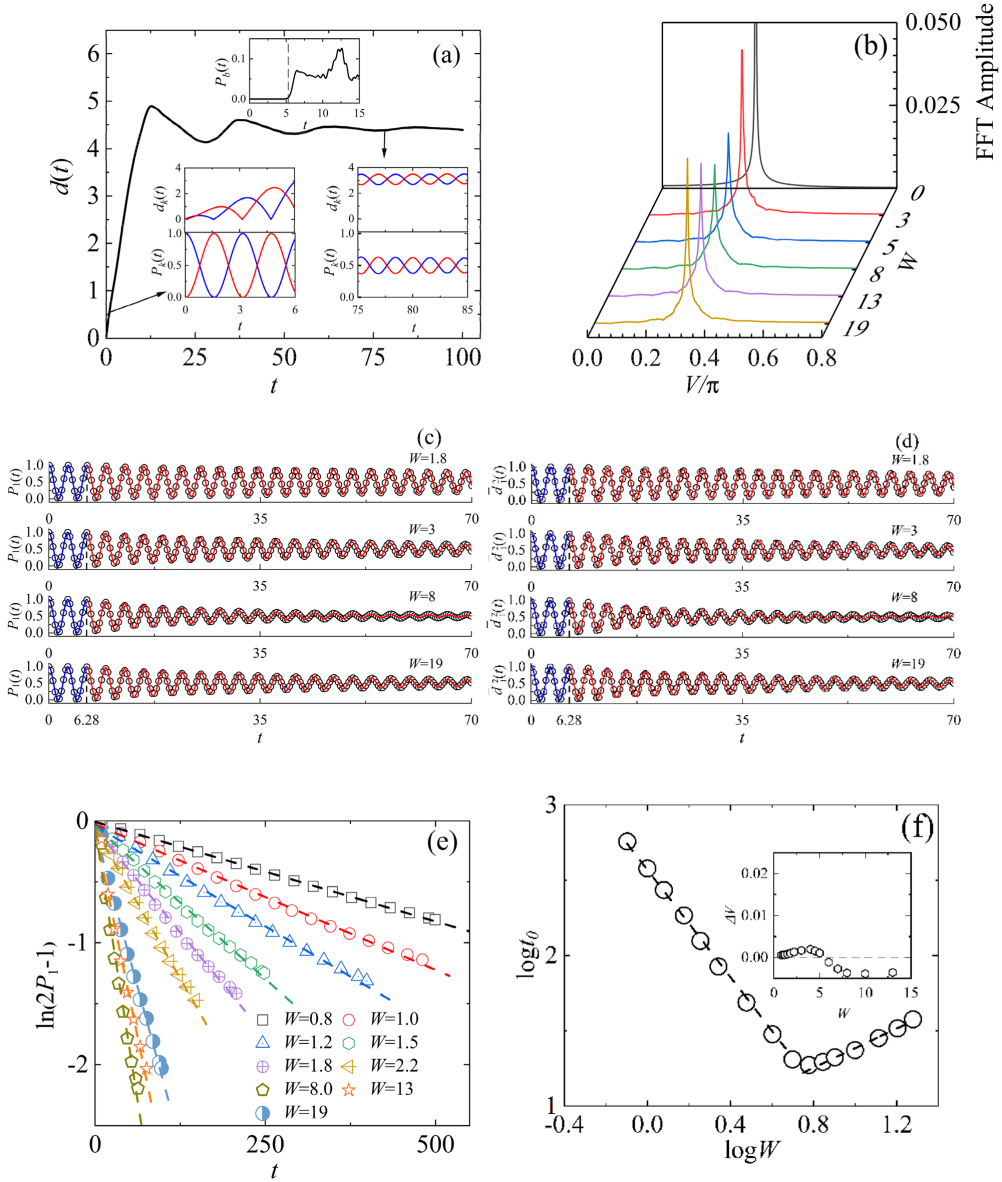
The electron oscillations in the edge-disordered bilayer graphene with $2N = {7688},{ }h = - 2.7,{ }u = 1$, $\varepsilon_{int}$ = 0, $\varepsilon_{out} \in \left[ { - W,W} \right]$. (**a**) The spreading width of the wavepacket $d\left( t \right)$ at $W$ = 3. The insets show the probability of finding electron at edge-sites $P_{b} \left( t \right)$, as well as the spreading width $d_{k} \left( t \right)$ and electron probability $P_{k} \left( t \right)$ in *k*-th layer, where *k* = 1 and 2 correspond to the blue and red lines, respectively. (**b**) FFT frequency spectrum of probability $P_{1} \left( t \right)$ as a function of $W$. (**c**) and (**d**) represent $P_{1} \left( t \right)$ and $\overline{d}_{1}^{2} \left( t \right)$ for $W = {1}{\text{.8}},{ 3}{\text{.0}},{ 8}{\text{.0}},{ 19}$ (open circles) and the corresponding fitting with Eqs. (14a), (18a) (blue lines) and Eq. (19)(red lines). (**e**) The numerical results of $\ln \left( {2P_{1} - 1} \right)$(symbols) and their corresponding linear fitting (dash lines) for different degrees of disorder *W.* (d) The values of $t_{0}$ versus *W* in log–log(symbols) and their corresponding linear fitting (dashed lines), indicating $t_{0} \sim W^{\alpha }$, with α = − 1.78 for W < 8, and α = 0.62 for W ≥ 8. The inset shows the values of $\Delta V$ versus *W*, where $\Delta V = V - V_{0}$.

The time evolution of the wavepacket in an edge-disordered bilayer graphene, as shown in Fig. 6, exhibits similar electron oscillations to those in bilayer square lattice. These oscillations can be described by Eqs. (14) and (18) before the wavepacket reaches the boundary, and by Eq. (19) after it reaches the boundary. Figure 6a illustrates the spreading width of the wavepacket $d\left( t \right)$ with $W$ = 3, which first exhibits linear growth with time and then ceases to grow after the wavepacket reaches the boundary. The insets show the time evolution of the probability of finding electrons at the edge sites $P_{b} \left( t \right)$, indicating the time when the wavepacket reaches the boundary at $t \approx 5$, as well as the spreading width $d_{k} \left( t \right){\text{ and}}$ electron probability $P_{k} \left( t \right)$ on the $k$-th layer, which indicate the decrease in the amplitude of the electron oscillations over time. The FFT frequency spectrum of $P_{1} \left( t \right)$, as shown in Fig. 6b, exhibits single-frequency signals for any degree of disorder $W$, and the signal strength undergoes a transition from decrease to increase at $W$ = 8. Figure 6c and d exhibit the oscillations of $P_{1} \left( t \right)$ and $\overline{d}_{1}^{2} \left( t \right)$ of typical disorder and their excellent fits. The fitting parameters for $W = {1}{\text{.8}},{ 3}{\text{.0}},{ 8}{\text{.0}},{ 19}$ are $t_{0} = {71}{\text{.115, 36}}{.112, 21}{\text{.858}}$ and $V = 1.0{0288, 1}{\text{.00531, 1}}{.00823}$. The fitting parameter $t_{0}$ is obtained from the linear fitting of the damped oscillation amplitudes ($t$ > 6.28), as shown in Fig. 6e, with the fit function $\ln (2P_{1} - 1) = t/t_{0}$. By substituting $t_{0}$ into Eq. (19) and then fitting the damped oscillation data of $P_{1} \left( t \right)$ and $\overline{d}_{1}^{2} \left( t \right)$, we can get the parameter *V*. From Fig. 6e we can see a significant reversal in the fitting slope at $W$ = 8. The relationship of $t_{0}$ and *V* versus *W* are shown in Fig. 6f, which clearly indicate that $t_{0} \sim W^{\alpha }$, with α = − 1.78 for $W$ < 8, and α = 0.62 for $W$ ≥ 8. The inset shows the parameter $V$ only undergoes a slight change with different degrees of disorder.

## Conclusions and discussions

In summary, this study finds that, in the coupled bilayer square lattices of edge disorder, the probability and spreading width of an electron wavepacket initially located on one layer exhibit periodic oscillations before the wavepacket reaches the boundary and become damped oscillations after the wavepacket reaches the boundary. As the disorder strength increases, the frequencies of these electronic oscillations remain almost unchanged, and the decay time of damped oscillations exhibits a transition from a decreasing behavior to an increasing behavior of decay time at a critical disorder value, which implies that the electron oscillations exhibit strong resistance against disorder perturbation in the regime of large disorder. We confirmed the universality of this anomalous damped oscillation behavior using bilayer graphene systems, emphasizing its independence from the geometric shape of the lattice. We note that, although damped periodic oscillations have been reported in the case of internal disorder in previous work^29,30^, it is unknown whether the oscillations under edge-disordered bilayer systems have the same form of damped oscillations. The unique aspect of this paper is that our numerical results reveal that the oscillations maintain the same form of damped oscillations under both small and large edge disorder. Particularly, there exists a crossover from a worse damped oscillation to an improved damped oscillation as $W$ increases, which is a new and unconventional phenomenon unreported in the literature. Since the site energies of edges are of the Anderson type of disorder given by $\left[ { - W,W} \right]$, the edges have both infinity walls and infinity wells, when $W$ goes infinity. Classically, some particles will be trapped in the wells, and some will bounce back and eventually will be trapped in the wells. Therefore, the phenomenon of the improved damped oscillations under large disorder is unexpected and anomalous from the classical point of view. Our results are significant in understanding the effects of edge-disorder on the stability of periodic electronic oscillations, which is important in designing novel quantum devices.

## Data Availability

The datasets used and/or analysed during the current study available from the corresponding author on reasonable request.

## References

[1] Novoselov KS, Mishchenko A, Carvalho A (2016). 2D materials and van der Waals heterostructures. Science.

[2] Novoselov KS, Geim AK, Morozov SV (2004). Electric field effect in atomically thin carbon films. Science.

[3] Yang M, Bin L, Shubin Y (2018). Ultrathin two-dimensional metallic nanomaterials. Mater. Chem. Front..

[4] Tan C, Cao X, Wu X-J (2017). Recent advances in ultrathin two-dimensional nanomaterials. Chem. Rev..

[5] Liu B, Zhou K (2019). Recent progress on graphene-analogous 2D nanomaterials: Properties, modeling and applications. Progress Mater. Sci..

[6] Mccann E, Fal'ko VI (2006). Landau-level degeneracy and quantum Hall effect in a graphite bilayer. Phys. Rev. Lett..

[7] Novoselov KS, Mccann E, Morozov S (2006). Unconventional quantum Hall effect and Berry’s phase of 2π in bilayer graphene. Nat. Phys..

[8] Zhang Y, Tang TT, Girit C (2009). Direct observation of a widely tunable bandgap in bilayer graphene. Nature.

[9] Cao Y, Fatemi V, Demir A (2018). Correlated insulator behaviour at half-filling in magic-angle graphene superlattices. Nature.

[10] Chen G, Sharpe AL, Gallagher P (2019). Signatures of tunable superconductivity in a trilayer graphene moiré superlattice. Nature.

[11] Liu Y-W, Su Y, Zhou X-F (2020). Tunable lattice reconstruction, triangular network of chiral one-dimensional states, and bandwidth of flat bands in magic angle twisted bilayer graphene. Phys. Rev. Lett..

[12] Cao Y, Park JM, Watanabe K (2021). Pauli-limit violation and re-entrant superconductivity in moiré graphene. Nature.

[13] Cao Y, Fatemi V, Fang S (2018). Unconventional superconductivity in magic-angle graphene superlattices. Nature.

[14] Banerjee S, Sardar M, Gayathri N (2005). Conductivity landscape of highly oriented pyrolytic graphite surfaces containing ribbons and edges. Phys. Rev. B.

[15] De Moura FA, Lyra MLJPRL (1998). Delocalization in the 1D Anderson model with long-range correlated disorder. Phys. Rev. Lett..

[16] Sedrakyan T (2004). Localization-delocalization transition in a presence of correlated disorder: The random dimer model. Phys. Rev. B.

[17] Zhao Y, Duan S, Zhang W (2012). (De) localization and the mobility edges in a disordered double chain with long-range intrachain correlation and short-range interchain correlation. J. Phys.: Condens. Matter.

[18] Zhao Y, Zhang W, Yan X-G (2020). Mobility edges and critical exponents in the disordered double chains with long-range correlation. Phys. E: Low-dimens. Syst. Nanostruct..

[19] Zuo ZW, Kang D (2022). Reentrant localization transition in the Su-Schrieffer-Heeger model with random-dimer disorder. Phys. Rev. A.

[20] Anderson PW (1958). Absence of diffusion in certain random lattices. Phys. Rev..

[21] Hu WM, Dow JD, Myles CW (1984). Effects of diagonal and off-diagonal disorder on the Anderson-model densities of states in two and three dimensions. Phys. Rev. B.

[22] Thouless DJ (1974). Electrons in disordered systems and the theory of localization. Phys. Rep..

[23] Abrahams EA, Anderson P, Licciardello D (1979). Scaling theory of localization: Absence of quantum diffusion in two dimensions. Phys. Rev. Lett..

[24] Patrick A, Lee TV (1985). Disordered electronic systems. Rev. Modern Phys..

[25] Lagendijk A, Tiggelen BV, Wiersma DS (2009). Fifty years of Anderson localization. Phys. Today.

[26] Punnoose A, Finkel'stein AM (2005). Metal-insulator transition in disordered two-dimensional electron systems. Science.

[27] Zhong J, Stocks GM (2007). Persistent mobility edges and anomalous quantum diffusion in order-disorder separated quantum films. Phys. Rev. B Condens. Matter.

[28] Fu L, Kane CL (2012). Topology, delocalization via average symmetry and the symplectic Anderson transition. Phys. Rev. Lett..

[29] Jiang JY, Lu YY, Wang C (2022). Periodic oscillation of quantum diffusion in coupled one-dimensional systems. Sci. China-Phys. Mech. Astron..

[30] Lu YY, Wang C, Jiang JY (2023). Periodic electron oscillation in coupled two-dimensional lattices. Chin. Phys. B.

[31] Zhong JX, Mosseri R (1995). Quantum dynamics in quasiperiodic systems. J. Phys. Condens. Matter.

[32] Moon BH (2021). Metal-insulator transition in two-dimensional transition metal dichalcogenides. Emerg. Mater..

[33] Moon P, Koshino M (2013). Optical properties of the Hofstadter butterfly in the moiré superlattice. Phys. Rev. B.

[34] Moon P, Koshino M, Son Y-W (2019). Quasicrystalline electronic states in 30∘ rotated twisted bilayer graphene. Phys. Rev. B.

